# Indoor heat measurement data from low-income households in rural and urban South Asia

**DOI:** 10.1038/s41597-022-01314-5

**Published:** 2022-06-09

**Authors:** Premsagar Tasgaonkar, Dipak Zade, Sana Ehsan, Ganesh Gorti, Nabir Mamnun, Christian Siderius, Tanya Singh

**Affiliations:** 1 WOTR Centre for Resilience Studies (W-CReS), Watershed Organisation Trust (WOTR), Pune, India; 2 School of Social Work, Tata Institute of Social Sciences (TISS), Mumbai, India; 3 Government College University Faisalabad, Faisalabad, Pakistan; 4 The Energy and Resources Institute (TERI), Delhi, India; 5 Department of Political Science and Institute of Behavioral Science - University of Colorado, Boulder, United States of America; 6 Bangladesh Centre for Advanced Studies (BCAS), Dhaka, Bangladesh; 7 Helmholtz Centre for Polar and Marine Research, Alfred Wegener Institute, Bremerhaven, Germany; 8 Wageningen Environmental Research, Wageningen, Netherlands; 9 Uncharted Waters, Sydney, Australia; 10 Climate Change Research Centre, University of New South Wales, Sydney, Australia

**Keywords:** Environmental impact, Environmental impact

## Abstract

Rising temperatures are causing distress across the world, and for those most vulnerable, it is a silent killer. Information about indoor air temperature in residential dwellings is of interest for a range of reasons, such as health, thermal comfort and coping practices. However, there have been only few studies that measure indoor heat exposure, and contrast these to outdoor temperatures in rural-urban areas, of which none are in South Asia. We aim to close this knowledge gap with our indoor and outdoor heat measurement dataset, covering five low-income sites in South Asia. Two sites are in rural areas (Maharashtra, India), while three sites focus on urban areas (Dhaka, Delhi and Faisalabad). Data are based on 206 indoor temperature data loggers and complemented by data from five outdoor automated weather stations. The data-set can be used to examine temperature and humidity variation in low-socioeconomic status households in rural and urban areas and to better understand factors aggravating heat stress. This is important to plan and implement actions for combating heat stress.

## Abstract (summary)

**Table:** 

Measurement(s)	air temperature • Humidity • atmospheric wind speed
Technology Type(s)	weather station • Temperature Sensor Device
Factor Type(s)	roof • wall • ventilation • room type • season • floor • ceiling • fan • direction • room • wind speed • solar radiation • hours • date • Evaporative cooler • Wall material
Sample Characteristic - Environment	weather
Sample Characteristic - Location	Jalna District • Yavatmal District • New Delhi • Faisalabad City • Dhaka

## Background & summary

Global warming is increasing the incidence, intensity, and duration of heat waves^1–4^. In the last decade, heat waves have caused more mortalities globally compared to any other climate-related disasters^5^. The deadly heatwave of 2015 in India and Pakistan, which lead to more than 3600 deaths^6^, has only confirmed the health threats emerging from heatwaves even for those accustomed to high temperatures.

The negative health impacts of longer and more frequent and intense heat waves will disproportionately affect the warmer and poorer regions of the world^7^. With one-fifth of the world population, South Asia could be one of the first densely populated places in the world to experience deadly heat waves with meteorological conditions exceeding survivability thresholds^8,9^. India, Bangladesh and Pakistan are projected to be severely affected by changes in weather patterns. Besides the direct impact on health, this could lead to an overall decrease in per capita consumption expenditure and gross domestic product^10^.

Weather parameters that define human thermal comfort and exposure to heat stress are air temperature, airflow (wind speed), humidity and radiation. Outdoor environmental factors that affect heat stress are degraded landscapes, low vegetation cover, and high settlement density^11^. In the indoor environment, factors influencing heat stress, in addition to outdoor weather parameters, are the building style, characterised by material type and construction of the roofs and walls, ventilation, internal and external shading, and the application of cooling devices (air conditioner, evaporative cooler or fan). The quantity of heat transmitted by, for example, different roof types determines the amount that is radiated inside and thus influences indoor temperature^12^.

Heat stress has various impacts on human health. Increased heat stress is generally associated with significant excess all-cause mortality^13,14^. Increased morbidity and occupational vulnerabilities are much more common. High temperatures are linked to multiple clinical syndromes such as heat stroke, heat exhaustion, heat syncope, and heat cramps^15^. Workers exposed to outdoor heat stress have reduced work productivity, experience physiological distress and adverse health impacts^16–18^. At night, high temperatures affect the quality of sleep, with prolonged exposure suggested to have significant impact on cognitive capacaties and people’s health. An increase in long term human migration has also been reported as a result of heat stress^19^.

Neighbourhood characteristics and properties of residential structures have a strong impact on heat exposure, heat stress and mortality. The poor and those from low socioeconomic status are more vulnerable to heat stress^20,21^. A study in South Africa found that living in wealthy areas and corresponding housing types may reduce heat related mortality by 50%^22^. In densely-built informal neighbourhoods in South Asia, exposure tends to be enhanced during night-time compared to exposure in green and open wealthier neighbourhoods^21^. Chronic heat stress, characterized by prolonged exposure also at night, as experienced by residents of informal settlements, is likely underestimated by studies using only outdoor weather station data^23^.

It is crucial to measure actual heat exposure of people in different indoor and outdoor settings to assess the relation between heat exposure and human health. Most epidemiological studies rely on meteorological data from standardised outdoor weather stations which may not accurately reflect personal heat exposure, such as inside houses or in areas with a landscape with very different characteristics^24^. A better understanding of indoor thermal conditions is essential because people spend most of their time in the indoor environment.

To fill this gap, a dataset of indoor temperature and relative humidity for low-income housing in five rural and urban sites has been created. Indoor temperature measurements were carried out in villages in Yavatmal and Jalna (Maharashtra, India), and the cities of Delhi (India), Dhaka (Bangladesh) and Faisalabad (Pakistan). These were supplemented by outdoor temperature and humidity measurements to gain better insight in temporal and spatial differences between indoor and outdoor temperature and humidity and the heat stress indicators that can be derived from these variables.

These measurements may help to understand indoor temperature variations in different housing types across various geographic locations in South Asia. Researchers, non-government organisations (NGOs)/civil societies working on public health issues, government departments (health, housing and disaster management authorities) and those engaged in policy formulations might find this dataset useful to plan and design actions for combating heat stress in indoor and outdoor environments. This dataset can be used to analyse the variations of temperature in different housing types and compare it with outdoor temperature. Those involved in the design and execution of Heat Action Plans and related heat warning systems can use the data to tailor warnings to expected indoor conditions in low-income settings which often differ from those observed outdoors.

## Methods

### General

Our indoor heat assessment is based on ambient temperature and relative humidity data. To complement the indoor data and provide direct comparison with outdoor temperature we have included outdoor observations from Automated Weather Stations (AWS) installed in the vicinity of the indoor study sites. An AWS is an automated version of the traditional weather station, and will typically consist of a weather-proof enclosure containing the data logger, rechargeable battery, optional telemetry to transfer data and the meteorological sensors with an attached solar panel or wind turbine and mounted upon a mast. These AWS measured air temperature (^0^Celsius), relative humidity (%), wind speed (km), and solar radiation. The period for urban indoor and outdoor measurements varied between the different study sites, but measurements were conducted mainly during the summer seasons of 2016 to 2018. Table 1 provides the details for each study site, data logger numbers and duration of data collection.

**Table 1:** Indoor and outdoor temperature measurements and measurement periods according to study sites.

Study sites	Sector	Number of indoor data loggers		Indoor measurement duration		Outdoor AWS measurement duration		Location of AWS	
		Installed	Functional till project end	From	To	From	To	Latitude	Longitude
Yavatmal, Maharashtra, India	Rural	20	20	May 2016	February 2018	May 2016	January 2018	20.424910	78.460935
Jalna, Maharashtra, India	Rural	17	16	March 2018	February 2019	March 2018	September 2018	20.335049	76.082572
Delhi, India	Urban	58	41	April 2016	October 2016	April 2016	February 2017	28.544600	77.147801
Faisalabad, Pakistan	Urban	48	34	March 2016	October 2016	April 2016	February 2017	31.4166	73.0707
Dhaka, Bangladesh	Urban	63	58	March 2016	October 2016	May 2016	October 2016	23.777176	90.399452

### Study areas and target population

**Fig. 1:**
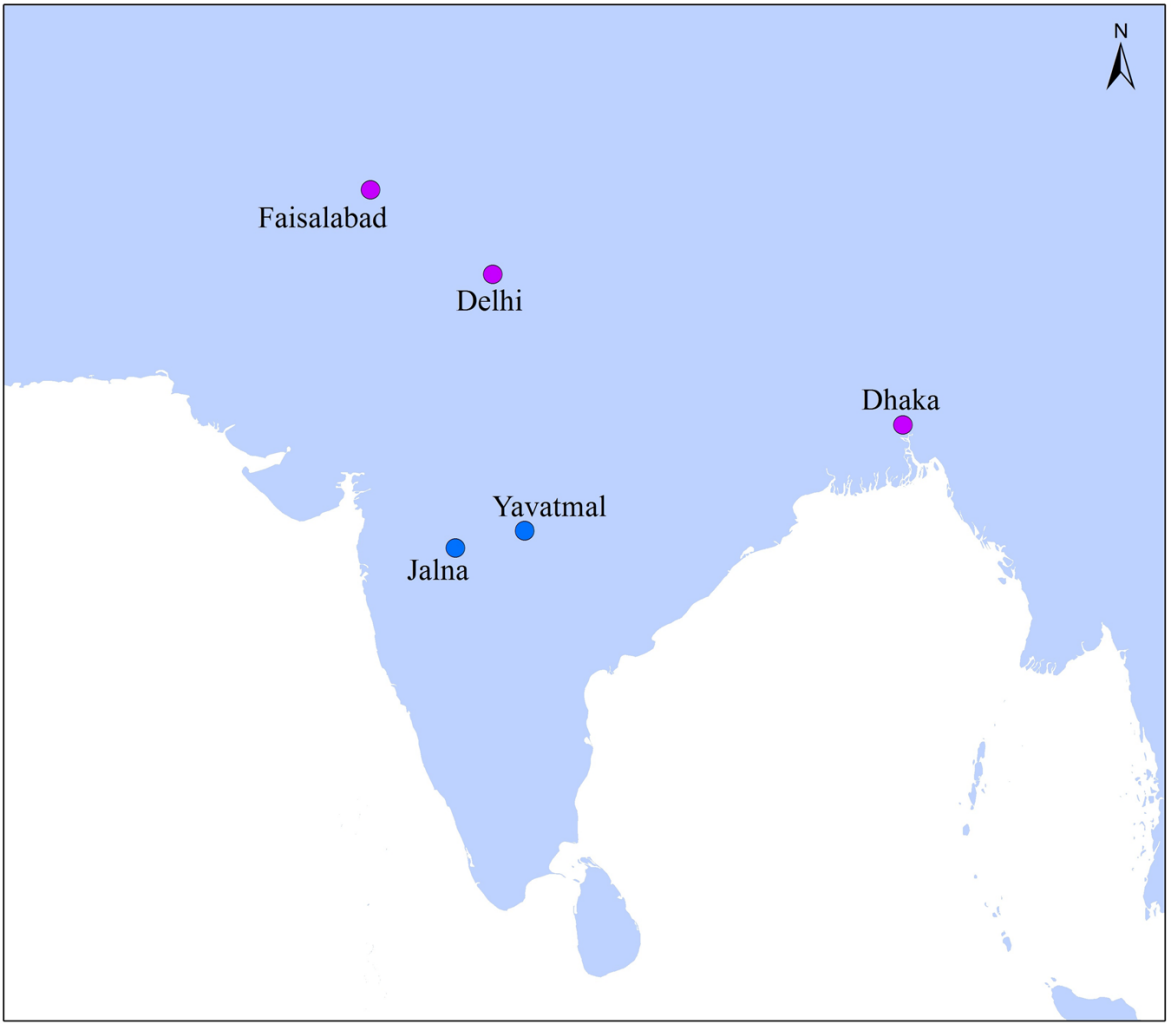
Case study sites, rural (blue) and urban (purple).

Indoor and outdoor observations were carried out in Delhi (India), and two villages in the Yavatmal and Jalna districts of Maharashtra (India), Dhaka (Bangladesh) and Faisalabad (Pakistan) (refer Fig. 1). Yavatmal and Jalna represent rural study sites while Delhi, Dhaka and Faisalabad are fast growing cities where temperatures are affected by the build-up surroundings, a process known as the urban heat island (UHI) effect. UHI is a phenomenon where urban areas are warmer than rural areas on average and this is often particularly pronounced during the evening and night^25^.

Most parts of South Asia suffer from spells of hot weather termed as heatwaves from March to June. April and May are known as the hottest month in the region. Yavatmal district is part of the Vidarbha region of Maharashtra, located in the central part of India. Many cities from this region feature among the hottest cities in the world. Jalna district is located in the central part of Maharashtra state in the northern Marathwada region. Recurrent droughts and parched landscapes characterise this region. The climate in both regions is classified as semi-arid, with hot and dry summers with relative humidity between 20% and 25% in the afternoon and moderately cold winters. The temperature starts rising from March till May, which is generally the hottest month of the year with a mean daily maximum temperature of about 42 °C in the Vidarbha region^26^ and up to 43 °C in the Marathwada^27^.

Delhi is the national capital of India and its second-most populous city, with about 29 million inhabitants. Of the nearly 1500 km^2^ total area, urban build-up areas cover about 700 km^2 ^^28^. The weather swings from hot and humid in summer to cold and dry in winter. During the summer months, the capital region’s daily maximum temperatures can rise to 40–45 °C, while in winters, daily minimum temperatures can drop to 2–3 °C^29^.

Faisalabad is the third-most-populous city in Pakistan with an area of 1300 km^2^ and a population of 3.4 million. The district is located in the semi-arid region, characterised by erratic rainfall^30^. The city experiences temperatures on both end of the spectrum, with summer temperature extremes as high as 50 °C and winter temperatures as low as −2 °C. The mean minimum and maximum temperatures in summer are about 27 °C and 39 °C^31^.

Dhaka, the capital of Bangladesh, covers an area of about 306 km^2^ and has a population of about 21 million inhabitants. The climate of Dhaka can be classified as a tropical savanna climate, with a distinct monsoonal season. The highest daily maximum temperatures are reached in March-May, rising to nearly 40 °C on individual days with the average being about 33 °C. Furthermore, a high average minimum temperature of over 26 °C is observed in the monsoon months June- August. These months are extremely humid, with an average evening relative humidity of about 75% and an average morning relative humidity of about 93%^21^.

The target populations in both the rural and urban areas were low-income households, a large socio-economic class in all three countries. India has the largest number of people (364 million people, 28% of the population) classified as multidimensional poor, as per the 2018 global Multidimensional Poverty Index (MPI)^23^. This index considers 11 indicators, spread across three dimensions of poverty- living standards, education and health. Pakistan and Bangladesh have similar percentages of poor people, and are ranked 4^th^ and 5^th^ globally.

The rural households were selected based on their economic status and the roof type of house. Roof type, along with wall and floor material, indicates the quality of houses and can be considered as an proxy indicator of socioecomic satus of a household^32,33^. The households were approached through local field staff- the *Wasundhara Sevaks*. Household members were informed about the study purpose after which consent was obtained via the household head. In the urban neighbourhoods of Delhi and Faisalabad, household heads were approached with support from the local community leaders. In Dhaka, residents appeared to be more accommodating and no mediatory support to gain household members’ trust was required. Urban households were selected based on two criteria; absence of any electric air-conditioning devices, and their location in selected low- and middle-income neighbourhoods. Air conditioning is generally related to higher socio-economic position in South Asia and often reported as an important socio-economic factor by which to classify vulnerable populations in heat stress studies^34,35^. The sampling of urban neighbourhoods was informed by a Local Climate Zone (LCZ) analysis in each city using Landsat images and spatial clustering^21^, with the final selection constrained by the practicalities of the research setup whereby indoor measurement locations had to be located near transects traversing these megacities (for details on the outdoor transect measurements, see Jacobs *et al*.^24^). While the indoor temperature and humidity data from various neighbourhoods can inform better heat warnings, the dataset does not have sufficient coverage to identify all particular areas of high or above average heat exposure across these cities. To design an effect early-warning system, a more elaborate observation campaign would be required.

Table 2 shows the descriptive statistics of indoor and outdoor temperature for each case study site. ‘Mean’, ‘min’ and ‘max’ were calculated by taking the mean of these respective values over all loggers in each site. ‘Min-min’(‘max-max’) is the absolute minimum (maximum) temperature measured indoors per site.

**Table 2:** Temperature ranges in the observational dataset per case study site.

	Delhi		Dhaka		Faisalabad		Yavatmal		Jalna	
	Mean	SD	Mean	SD	Mean	SD	Mean	SD	Mean	SD
Mean	32.0	(1.4)	30.9	(1.4)	32.7	(3.5)	28.9	(4.6)	28.8	(4.3)
Min	26.5	(1.8)	27.0	(1.8)	27.9	(3.3)	23.1	(4.6)	23.6	(4.3)
Max	41.2	(3.3)	39.2	(2.9)	37.1	(3.8)	38.4	(6.4)	37.3	(5.2
Min-Min	21.6	—	18.2	—	17.3	—	11.8	—	12.9	—
Max-Max	48.7	—	44.4	—	44.9	—	53.8	—	47.8	—

The demographic details of the households where indoor data loggers were installed are shown in Table 3. Family size is largerst in Faisalabad where extended families live in small two-rooms houses. Households in Delhi and Dhaka had about 50% more men than females, of working age, which is likely an effect of labour migration whereby the family stays behind in rural villages.

**Table 3:** Site-wise household demographic details.

Characteristics	Delhi	Dhaka	Faisalabad	Yavatmal	Jalna
Total households (data logger installed)	58	63	48	20	17
Number of households for which socio-economic data is available	39	54	39	20	17
Average family size	5	4	7	5	5
Average number of rooms	2	2	2	4	3
Sex					
Male	113	143	123	48	43
Female	83	96	130	51	41
Total members	196	239	253	99	84
Total number of individuals (in %) as per age-group (in years)					
Up to 5	9%	11%	16%	4%	12%
6 to 10	9%	10%	13%	7%	8%
11 to 15	8%	8%	9%	8%	0%
16 to 25	27%	18%	14%	8%	18%
26 to 35	25%	25%	18%	35%	27%
36 to 45	13%	13%	15%	13%	7%
46 to 55	5%	10%	5%	10%	12%
56 to 65	2%	4%	2%	8%	14%
66 Above	3%	1%	8%	6%	1%

Heat stress was recognized as a health risk during the summer period in the majority of households in both urban and rural sites. Table 4 presents the top four perceived Heat Related Symptoms (HRS) and coping strategies by study sites. In the urban sites, feverish conditions/ undiagnosed fever is an often reported HRS followed by dehydration (including diarrhoea and vomiting). Tiredness and exhaustion was particularly often mentioned in Dhaka. Increased fluid intake, taking more showers and increased use of evaporatice coolers were viewed as key coping strategies. In rural sites, heavy sweating, leg cramps, thrist and fatigue are the most reported HRS, probably because people here are mostly engaged in outdoor activities during summer. Drinking more water, seeking shade, and covering the head when outdoors were key coping strategies reported.

**Table 4:** Heat related symptoms and coping strategies, collected through an interview with household heads using an open-ended questionnaire.

Study sites	Top four perceived Heat Related Symptoms	Percentage	Top 4 heat coping strategies	Percentage
Delhi (N = 55)	Feverish conditions /Undiagnosed fever	20%	Increase fluid intake	42%
	Dehydration (including diarrhoea and vomiting)	13%	Taking more showers	24%
	Heatstroke	5.5% (diagnosed)	Use active cooling, like evaporative coolers, more often	18%
		5.5% (self-reported)		
	General weakness	4%	Sleep in open/cooler places of the house	11%
Dhaka (N = 58)	Tiredness/feeling exhausted	90%	Go to cooler places	86%
	Feverish conditions	72%	Resting more	57%
	Diarrhoea	26%	Staying hydrated	36%
	Skin rash	21%	Using hand fans, using wet cloths, sleeping on floor	22%
Faisalabad (N = 47)	Feverish conditions	60%	Taking more showers	47%
	Diarrhoea	49%	Staying hydrated	40%
	Headache	47%	Resting more	23%
	Dehydration	32%	Go to cooler places	13%
Yavatmal (N = 20)	Heavy Sweating	51%	Drinking more water	81%
	Leg Cramps	52%	Covering head during work	66%
	Intense Thirst	60%	Desert cooler	67%
	Fatigue	37%	Ceiling fans	76%
	Dry Mouth	25%	Table fans	40%
	Headache	27%	Changed cooking times (cooking earlier)	75%
Jalna (N = 17)	Headache	23%	Seeking shade, using light colored clothing or covering head with a cloth - while working outdoor	90%
	Heavy Sweating	21%	Changed cooking times (cooking earlier) in summers	60%
	Fatigue	20%	Ceiling fans	67%
	Intense Thirst	19%	Table fans	14%
	Leg Cramps	9%		

### Ethical considerations

The study design complied with all relevant international and national ethical regulations, having been approved by the International Development Research Centre (IDRC), Canada, whose ethics guidelines follow The 2^nd^ edition of the Canadian Tri-Council Policy Statement on Ethical Conduct of Research Involving Humans. Informed consent was acquired from all research participants. Link to the ‘The 2^nd^ edition of the Canadian Tri-Council Policy Statement on Ethical Conduct of Research Involving Humans’: https://publications.gc.ca/site/eng/381622/publication.html

### Instruments

#### Indoor measurements

With new technological developments, measuring indoor meteorological parameters without interfering with household activities has become much easier. Data loggers are miniaturised electronic devices used to measure indoor temperature and relative humidity. Here, HOBO UX 100-001 and HOBO UX100-011 data loggers were installed in houses within each study site. The indoor data loggers measured air temperature (^0^Celsius) (HOBO UX 100 001) and relative humidity (%) (HOBO UX100-011) with measurement intervals of 10 minutes.

The researchers and field staff installed these data loggers in the houses. In total, 206 temperature data loggers were installed across the study sites. Twenty of these loggers were able to measure relative humidity in addition to air temperature. In urban sites, six of these were installed to capture variations in humidity between different neighbourhoods, with an additional two installed in the rural study sites. Out of the initially installed data loggers, 169 remained in operation throughout the season and provided a time series of sufficient length. Some loggers stopped functioning, and in several cases occupants migrated. Others were considered faulty after the checking of the data, with records showing abnormal jumps in temperature possibly due to the logger being moved around. In few cases, loggers were thrown away due to mistrust by household members. Houses built in low-income neighbourhoods, especially in urban slums, often lack official status and house owners or renters treat outsiders with caution. In Faisalabad, Pakistan, particularly, concerns about the device being used for other purpose than temperature measurements had to be negotiated. Within the urban settings of Delhi, Dhaka and Faisalabad, indoor data loggers were installed in different neighborhoods, as shown in Table 5. In both rural areas, loggers were clustered in a single village.

**Table 5:** Overview of the total number of data loggers in each urban study sites aggregated per neighbourhood.

Delhi	Data loggers	Dhaka	Data loggers	Faisalabad	Data loggers
Kasturbha Nagar	11	Chala/Badda	15	Babu Wala	14
Laxmi Nagar	08	East Nakhalpara	06	Clock Tower	04
Lodhi Colony	11	Korail Basti	14	D –Block	11
Patparganj slum	07	Kunipara/Tejgaon	11	Gulamabad	09
Railway Colony	10	T and T colony	07	Murad Abad	14
Sarai Kale Khan	12	Wireless Gate	07	—	—

For installing the data loggers, the research team followed a protocol, pre-tested in the Faisalabad study site, to minimise random measurement variations. The data loggers were installed either in the bedrooms or living rooms of households. In several cases, these two types of rooms were not distinguishable, as households with a lower socioeconomic background lack space. Some households even have the kitchen, bedroom and living room all in one room. In these houses, we installed the logger close to the sleeping corner. Otherwise, the decision where to install them was based on where household members spent most of their time. All loggers were fixed to a wall or a piece of furniture at approximately 1.3 m from the floor, away from any heat or cold sources, and from any solar radiation. Monthly to two-months visits were made to the houses to check on the loggers. During periodic field visits, temperature data were transferred to a laptop, checked and added to the database.

#### Outdoor measurement

One AWS was installed in each of the five sites to determine the temporal evolution of the weather. An AWS is an assembly of various sensors to monitor various environmental parameters. The typical key sensors used in a weather station and collected in this study are air temperature, humidity, wind direction and speed (anemometer), global radiation, and evaporation. All AWS (Wireless Vantage Pro2 Plus or equivalent, Davis Instruments, USA) came with a small 5-watt solar panel which powered the system. These weather stations promise reliable operation without any manual intervention and the data can be accessed using the internet and a mobile application. All AWS were programmed to record data in an hourly interval.

The AWS stations were installed in an open space with sufficient distance from any physical structure to avoid direct shading, and away from large water bodies, industrial heat sources or high tension cables (both overhead and underground). In the two rural sites, the AWSs were placed close to the village, but at 300 m from the nearest building. In urban areas, buildings could not be avoided within a similar radius, but since our aim was to capture typical urban conditions, this was considered acceptable^36^. They were fenced for protection from humans, stray dogs and livestock. In Faisalabad, the AWS was installed at the campus of Government University College Faisalabad (GCUF), situated close to the city center. In Delhi, it was installed in front of The Energy and Resources Institute (TERI) University campus in South Delhi. In Dhaka, the AWS was installed close to the Bangladesh Meteorological Institute office building. In all the sites, prior permission for installation was obtained from the concerned authorities. GCUF and TERI were both project collaborators. All AWS stations were installed by experienced meteorologists following the user guidelines provided by the manufacturer. More detail on the AWS installation can be found in the supplement file (README FILE 4).

#### Data on housing characteristics

To relate indoor heat exposure with housing characteristics, data was collected on roof and wall material, roof and wall colour, the orientation of windows, doors and ventilation holes, room type and room height and presence of fans and evaporative coolers (Table 6). All houses are low-income housing types and are made of low cost and locally available materials, with some basic ventilation in the form of air holes, but hardly any specific insulation other than provided by the type of material itself which is indicated in the data tables, e.g. for walls and roofs. None of these houses had specific air sealing measures. Characteristics can be grouped into two; i. construction properties, such as roof and wall type; and ii. active and passive ventilation, e.g. ceiling fans or evaporative coolers, and the direction of ventilation and whether they were opened at night and closed during the day.

**Table 6:** Housing characteristics.

Characteristics	Delhi	Dhaka	Faisalabad	Yavatmal	Jalna
	(58 Houses)	(63 Houses)	(48 Houses)	(20 Houses)	(17 Houses)
Roof type					
Galvanized iron/metal sheets	22%	60%	—	60%	52%
RCC slab/concrete	43%	30%	15%	35%	41%
Stone/slate	15%	—	—	—	—
Tiles	—	—	69%	5%	—
Floor above*	20%	10%	13%	—	—
Straw/thatch	—	—	2%	—	6%
Wall type					
Concrete	8%	—	—	—	—
Bricks	92%	—	—	65%	76%
Single Brick	—	65%	19%	—	—
Double Brick	—	—	81%	—	—
Brick and mud	—	—	—	35%	18%
Tin sheet	—	35%	—	—	6%
Fan					
Ceiling	73%	77%	100.00%	85%	82%
Wall	5%	—	—	—	—
Both	2%	—	—	—	—
Standing/Table	7%	20%	—	15%	18%
No	14%	3%	—	—	—
Evaporative cooler					
Yes (including desert cooler)	53%	—	10%	100%	18%
No	47%	100%	90%	—	82%
Ventilation (day)					
No ventilation	3%	3%	4%	5%	0%
Ventilation only in one side of the house	32%	17%	63%	60%	41%
Ventilation perpendicular	17%	3%	10%	10%	18%
Cross ventilation	47%	77%	23%	25%	41%
Ventilation (night)					
No ventilation	12%	8%	10%	16%	35%
Ventilation only in one side of the house	54%	15%	65%	9%	24%
Ventilation perpendicular	5%	10%	8%	21%	18%
Cross ventilation	29%	67%	17%	53%	24%

*Floor above- refers to apartments that have another apartment (floor) on top and hence they do not have a roof exposed to the sun.

## Data Records

The main data-set is stored in a data repository as standalone datafiles (to access: 10.6084/m9.figshare.12546368.v1)^37^. The data can be categorised into three types i) indoor temperature and humidity data; ii) outdoor temperature data and other weather parameters, such as humidity, rainfall, solar radiation and wind speed and direction; iii) housing characteristics information. Indoor and outdoor temperature and humidity data is stored in.CSV file format (Comma-Separated Values). The housing characteristics can be found in the household data sheet (HSD) in a.CSV file format for each study site. In addition, the repository also contains a sample copy of the consent form.

## Technical Validation

In all houses, HOBO UX100-001/011 temperature data loggers from the Onset Corporation were installed. The temperature accuracy for these loggers is ± 0.21 K from 0° to 50 °C. After purchase, all loggers were validated in a climate chamber against a certified calibrated data logger. None of the loggers deviated more than ± 0.15 °C from the certified sensor. The accuracy of the loggers measuring relative humidity in addition to temperature, HOBO UX100-011, is ± 2.5% in a relative humidity range between 10% to 90% for humidity. The technical specifications of the data loggers are summarised in Table 7.

**Table 7:** Physical and technical specification parameters of the temperature and humidity data logger.

Parameter	Temperature	Humidity
Data logger type (Sensor)	HOBO UX100-001*	HOBO UX100-011**
Temperature/humidity range	−20° to 70 °C	1% to 95% (non-condensing)
Accuracy	± 0.21 °C from 0° to 50 °C	±2.5% from 10% to 90% typical to a maximum of ± 3.5% including hysteresis at 25 °C; below 10% and above 90% ± 5% typical
Resolution	0.024 °C at 25 °C	0.01%
Memory size	128 KB (84,650 measurements, maximum)	128 KB (63,488 measurements, maximum)
Size	3.66 × 5.94 × 1.52 cm	3.66 × 8.48 × 2.29 cm
Weight	23 g	30 g
Temperature parameter	°C	°C

Note: *measures temperature only; **measures temperature and relative humidity.

The following three steps ensured the technical validation and quality control of the data. First, the data loggers’ performance was checked after four to five days of installation to ensure that the data loggers were recording correctly. Second, in few cases, cross verification measurements of temperatures were taken during household interviews using a Kestrel-4600 handheld device. The handheld device can measure air temperature, wind speed, black globe temperature, and relative humidity. Every unit of this device is factory-tested for measurement accuracy and waterproof integrity. We did not find any difference between temperatures recorded by the handheld device and with the data loggers. Third, all data were downloaded from the data loggers into a laptop using the HOBO UX software and applying the unit in degree Celsius (°) for temperature and relative humidity (%) for humidity. Then the data were read out into.CSV format and merged into one.CSV file.

The station (Wireless Vantage Pro2 Plus, Davis Instruments, USA) includes solar radiation sensors and a daytime fan-aspirated radiation shield for temperature and humidity devices. Furthermore, wind speed and wind direction are measured using a cup anemometer and wind vane, respectively. The station is also equipped with a rain gauge and a barometer. All sensors measure at an interval of one minute and the readings are subsequently averaged or summed to obtain hourly values, with accuracies for wind speed 0.5 m s-1, air temperature 0.5 °C, relative humidity 2%, pressure 1hPa, rainfall 4% for rates up to 100 mm hr-1, solar radiation 5%. No household in the rural sites was more than 5 miles from the weather station. Urban households were within a 10 miles radius from the installed weather station.

The housing characteristics questionnaire was designed based on the research questions of the study project. To ensure that the questions are relevant and meaningful, pre-testing of the questionnaire (pre-coded and open-ended) was conducted in the study area through pilot surveys. After pre-testing, a revised tool was used for the data collection. During the survey, a researcher checked all the completed housing characteristics questionnaires for completeness (no missing values and units) and accuracy before they were submitted for data entry.

## Usage Notes

This dataset is anonymised and cannot be used to link data with individual study participants. All data (indoor and outdoor temperature) is stored into a.CSV file. It includes the actual readings from the data loggers and from the AWS station, in a cleaned and arranged format. We have also provided data supplement files (as README FILES) providing information on housing characteristics (README FILE 1), installation information on the indoor data loggers (README FILE 2), AWS parameters (README FILE 3) and AWS installation manual (README FILE 4). These supplemental files are available in a readme file format. The datasets can be used for indoor and outdoor temperature analysis for any region and/or to investigate urban-rural temperature variation. For example, there are a range of variables that will allow the user to examine factors that affect indoor temperature such as different roofing structures, wall structure and cooling devices. More information on the data format, labels and details can be obtained from a readme file provided in the data records link.

Users can download the indoor and outdoor temperature dataset and select the study areas (Jalna, Yavatmal, Delhi, Faisalabad and Dhaka) separately as per their analysis requirement. This dataset is complete and will not change later. In order to download data there is no need to take permission of the authors. As such, we encourage readers to use the data from the Figshare data repository.

**Fig. 2:**
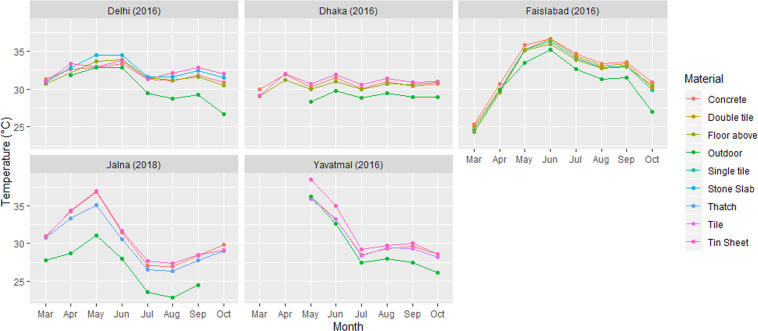
Monthly average indoor-outdoor temperature (°C).

As an illustration, we present a site-wise analysis of monthly average indoor-outdoor temperature variations (Fig. 2). Mean monthly temperatures, here clustered by roofing structure type, were obtained by aggregating temperature of 10-minute intervals into monthly average temperature. Mean monthly indoor temperatures are higher than temperatures outdoors, for all roof structure types. Indoor temperatures generally follow outdoor temperatures during the day, but houses cool down slower during the evening and night, so mean temperature indoors exceed those measured outdoors.

The effect of housing characteristics differs per site and over the summer season. In Delhi, in summer, the highest monthly mean temperature was recorded in stone roof houses (34.5 °C). In houses with these types of roofs, indoor mean monthly temperatures are higher than the outdoor temperature during late summer, in May and June. In early summer, in April, the indoor temperature in tin roof houses (33.4 °C) is higher than outdoor temperature (32.8 °C). In Dhaka the highest monthly mean temperature was recorded in tin roof houses in the month of June (31.9 °C). The indoor temperature in tile roof houses was lower than in houses with a tin or concrete roof.

In Faisalabad, temperatures fluctuate strongly over the season, with the average highest outdoor temperature was recorded in June. Houses with a roof made of “concrete” slabs record the highest indoor temperatures, though not much different from those in houses with a single tile and double tile roofs houses. Poorer neigbourhoods in Faisalabad generally have few houses with tin roofs and none was included in our sample.

The indoor temperature observation in the rural site Yavatmal ranged from 36 °C to 38 °C in tin roof houses, while in concrete houses it was in the range between 34 °C to 36 °C. The monthly temperature variation in tin roof houses was higher by 3 °C to 4 °C than for outdoor temperature in May and June 2016. In the other rural study site, Jalna, the indoor temperature in thatch roof houses was lower (35.1 °C) than in concrete (36.8 °C) and tin roof (36.9 °C) houses. The recorded temperature shows that throughout the study period, the temperature in tin roof houses in rural areas was higher than in concrete roof houses, and even higher than the outdoor temperature. It is observed that when the outdoor temperature reaches its maximum, the low-mass corrugated metal roofs cool down rather quickly in the evenings compared to houses made out of concrete, whereas other roofs do not react similarly.

**Fig. 3:**
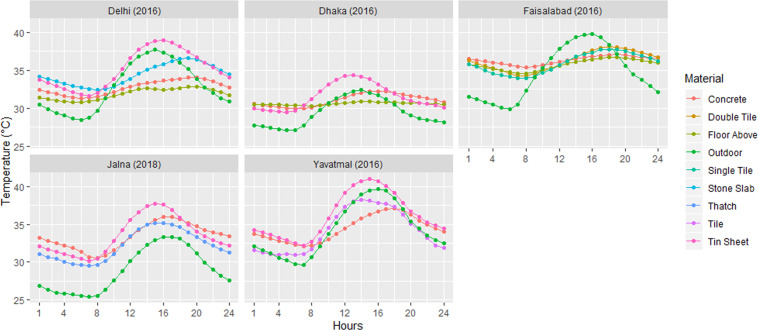
Indoor-outdoor diurnal temperature variation for the summer months of May and June.

Figure 3 highlights the diurnal temperature variation for the five study sites. All sites except Dhaka, which has many overcast days during summer, show a strong diurnal pattern in termperatures, with daily maxima reaching up to or even over 40 °C both indoors and outdoors. In Delhi and Faisalabad, the ‘floor above’ houses are cooler than the outdoor. During noon, the indoor temperature in tin roofed houses (Yavatmal, Jalna, Delhi and Dhaka) is higher than in houses with other roof types. In Faisalabad, the average outdoor temperature is higher than all type of houses from 11 am to 6 pm. It is also observed that the outdoor temperature starts decreasing from 8 pm but the indoor temperature remains high. At night, indoor temperatures are generally higher than outdoor temperatures as heat is trapped inside the houses and within the buildup infrastructure in densily populated neighbourhoods.

## Data Availability

This study did not use any computer codes to generate the indoor and outdoor temperature dataset. Online, code is available to calculate various heat indices such as the Wet Bulb Globe Temperature (WBGT)^38^ based on the collected data.

## References

[1] Perkins-Kirkpatrick SE, Lewis SC (2020). Increasing trends in regional heatwaves. Nat. Commun..

[2] Perkins, S. E., Alexander, L. V & Nairn, J. R. Increasing frequency, intensity and duration of observed global heatwaves and warm spells. *Geophys. Res. Lett*. **39**, (2012).

[3] Argüeso D, D Luca A, Perkins‐Kirkpatrick SE, Evans JP (2016). Seasonal mean temperature changes control future heat waves. Geophys. Res. Lett..

[4] Brown SJ (2020). Future changes in heatwave severity, duration and frequency due to climate change for the most populous cities. Weather Clim. Extrem..

[5] International Federation of Red Cross and Red Crescent Societies (IFRC). *Tackling the humanitarian impacts of the climate crisis together*. *World Disaster Report 2020* (2020).

[6] Wehner MF, Stone DA, Krishnan H, Achuta Rao K, Castillo F (2016). The deadly combination of heat and humidity in India and Pakistan in summer 2015. Bull. Am. Meteorol. Soc..

[7] Gasparrini A (2017). Projections of temperature-related excess mortality under climate change scenarios. Lancet Planet. Heal..

[8] Im E-S, Pal JS, Eltahir EAB (2017). Deadly heat waves projected in the densely populated agricultural regions of South Asia. Sci. Adv..

[9] McKinsey Global Institute. *The Future of Asia: Climate risk and response in Asia*. www.mckinsey.com/mgi. (2020).

[10] Mani, M., Bandyopadhyay, S., Chonabayashi, S., Markandya, A. & Mosier, T. *South Asia’s hotspots: the impact of temperature and precipitation changes on living standards*. 10.1596/978-1-4648-1155-5 (World Bank Group, 2018).

[11] Harlan SL, Brazel AJ, Prashad L, Stefanov WL, Larsen L (2006). Neighborhood microclimates and vulnerability to heat stress. Soc. Sci. Med..

[12] Ponni M, Baskar R (2014). Evaluation of indoor temperature through roof and wall temperatures—an experimental study in hot and humid climate. Int. J. Eng. Innov. Technol.(IJEIT).

[13] Azhar, G. S. *et al*. Heat-related mortality in India: excess all-cause mortality associated with the 2010 Ahmedabad heat wave. *PLoS One* **9** (2014). 10.1371/journal.pone.0091831 PMC3954798 24633076

[14] Ingole V, Rocklöv J, Juvekar S, Schumann B (2015). Impact of heat and cold on total and cause-specific mortality in Vadu HDSS—a rural setting in Western India. Int. J. Environ. Res. Public Health.

[15] Kovats RS, Hajat S (2008). Heat stress and public health: a critical review. Annu. Rev. Public Heal..

[16] Kjellstrom T, Holmer I, Lemke B (2009). Workplace heat stress, health and productivity-an increasing challenge for low and middle-income countries during climate change. Glob. Health Action.

[17] Tawatsupa B, Lim L-Y, Kjellstrom T, Seubsman S, Sleigh A (2010). The association between overall health, psychological distress, and occupational heat stress among a large national cohort of 40,913 Thai workers. Glob. Health Action.

[18] Venugopal V, Chinnadurai JS, Lucas RAI, Kjellstrom T (2015). Occupational heat stress profiles in selected workplaces in India. Int. J. Environ. Res. Public Health.

[19] Mueller V, Gray C, Kosec K (2014). Heat stress increases long-term human migration in rural Pakistan. Nat. Clim. Chang..

[20] Leichenko R, Silva JA (2014). Climate change and poverty: vulnerability, impacts, and alleviation strategies. Wiley Interdiscip. Rev. Clim. Chang..

[21] Jacobs C (2019). Patterns of outdoor exposure to heat in three South Asian cities. Sci. Total Environ..

[22] Scovronick N, Armstrong B (2012). The impact of housing type on temperature-related mortality in South Africa, 1996–2015. Environ. Res..

[23] Ramsay EE (2021). Chronic heat stress in tropical urban informal settlements. Iscience.

[24] Jacobs, C., Siderius, C. & Tasgaonkar, P. *Combating heat stress in south Asia-a cross-CARIAA effort: final technical report on IDRC project no.–108317-001*. (2020).

[25] Oke TR (1982). The energetic basis of the urban heat island. Q. J. R. Meteorol. Soc..

[26] Government of Maharashtra. Yavatmal district information- Official webpage. https://yavatmal.gov.in/ (2021).

[27] Government of Maharashtra. About Jalna District. https://jalna.gov.in/about-district/demography/ (2020).

[28] India Census. Delhi Population 2011–2021. (2021).

[29] Sati AP, Mohan M (2018). The impact of urbanization during half a century on surface meteorology based on WRF model simulations over National Capital Region, India. Theor. Appl. Climatol..

[30] Kashif, F. S., Salik, K. M. & Ishfaq, S. Climate induced rural-to-urban migration in Pakistan. (2016).

[31] Khan M, Shahid Khadim H (2015). A study of air pollution and human health in Faisalabad city. Pakistan. Int. J. Core Eng. Manag..

[32] Mohanty SK (2009). Alternative wealth indices and health estimates in India. Genus.

[33] Siegert K (2021). The effect of socioeconomic factors and indoor residual spraying on Malaria in Mangaluru, India: a case-control study. Int. J. Environ. Res. Public Health.

[34] Mastrucci A, Byers E, Pachauri S, Rao ND (2019). Improving the SDG energy poverty targets: residential cooling needs in the Global South. Energy Build..

[35] Tan J (2007). Heat wave impacts on mortality in Shanghai, 1998 and 2003. Int. J. Biometeorol..

[36] Oke, T. R. *Initial guidance to obtain representative meteorological observations at urban sites, Report no. 81*. (2004).

[37] Tasgaonkar, P. Indoor heat measurement data from low-income households in rural and urban South Asia, *figshare*, 10.6084/m9.figshare.12546368.v1 (2022). 10.1038/s41597-022-01314-5 PMC9184534 35680940

[38] Liljegren JC, Carhart RA, Lawday P, Tschopp S, Sharp R (2008). Modeling the wet bulb globe temperature using standard meteorological measurements. J. Occup. Environ. Hyg..

